# Mapping Quantitative Trait Loci Associated with Toot Traits Using Sequencing-Based Genotyping Chromosome Segment Substitution Lines Derived from 9311 and Nipponbare in Rice (*Oryza sativa* L.)

**DOI:** 10.1371/journal.pone.0151796

**Published:** 2016-03-24

**Authors:** Yong Zhou, Guichun Dong, Yajun Tao, Chen Chen, Bin Yang, Yue Wu, Zefeng Yang, Guohua Liang, Baohe Wang, Yulong Wang

**Affiliations:** 1 Jiangsu Key Laboratory of Crop Genetics and Physiology/Co-Innovation Center for Modern Production Technology of Grain Crops, Key Laboratory of Plant Functional Genomics of the Ministry of Education, Yangzhou University, Yangzhou 225009, China; 2 Lixiahe Agricultural Research Institute of Jiangsu Province, Yangzhou 225007, China; Aberystwyth University, UNITED KINGDOM

## Abstract

Identification of quantitative trait loci (QTLs) associated with rice root morphology provides useful information for avoiding drought stress and maintaining yield production under the irrigation condition. In this study, a set of chromosome segment substitution lines derived from 9311 as the recipient and Nipponbare as donor, were used to analysis root morphology. By combining the resequencing-based bin-map with a multiple linear regression analysis, QTL identification was conducted on root number (RN), total root length (TRL), root dry weight (RDW), maximum root length (MRL), root thickness (RTH), total absorption area (TAA) and root vitality (RV), using the CSSL population grown under hydroponic conditions. A total of thirty-eight QTLs were identified: six for TRL, six for RDW, eight for the MRL, four for RTH, seven for RN, two for TAA, and five for RV. Phenotypic effect variance explained by these QTLs ranged from 2.23% to 37.08%, and four single QTLs had more than 10% phenotypic explanations on three root traits. We also detected the correlations between grain yield (GY) and root traits, and found that TRL, RTH and MRL had significantly positive correlations with GY. However, TRL, RDW and MRL had significantly positive correlations with biomass yield (BY). Several QTLs identified in our population were co-localized with some loci for grain yield or biomass. This information may be immediately exploited for improving rice water and fertilizer use efficiency for molecular breeding of root system architectures.

## Introduction

Rice (*Oryza sativa* L.) is one of the most important food sources. With the booming population around the world, we have to produce 40% more rice to reduce the food crisis [1]. Rice has the greatest water requirement of all cereal crops, requiring 3000~5000 liters of water per kilogram of grain produced in flooded fields. Rice plants often experience drought in environments when rainfall is not sufficient to maintain flooded paddy conditions. As an important organ of the plant, the roots are involved in the acquisition of water and nutrients, and in the synthesis of plant hormones [2]. In previous studies, a strong correlation was found between root morphology and grain yield or biomass yield [3,4]. Therefore, study on rice root is of meaningful. Root morphology breeding is thought to be an important strategy to achieve a new breakthrough of rice high breeding in the future [5].

Root morphology includes root length, root number, root thickness, root weight, root vitality and total absorption area, etc. All these physiological and morphological traits of roots affect shoot growth [6]. For example, the maximum root length determines the efficiency of water and nutrition uptake, while root number, root thickness, and root length density determine the intensity of colonization of the soil profile [7]. Generally speaking, thick roots can decrease the risk of cavitations and facilitate water flux [8]. Several studies indicated that root biomass is strongly correlated with aboveground biomass [2]. Root oxidation activity is regarded as an important index of root physiological activity [2,9,10]. Root vitality represents the strength of metabolism, which further determines the growth of leaf and the level of grain yield, and root total absorption area reflects the ability of nutrition utilization. As a result, the rice root traits have been widely studied from the perspective of genetics and physiology.

Mutants of root traits are well materials for study on root development. Genetic approaches in a series of root mutants, such as crl1, crl4/Osgnom1, wox11, Oscand1, and Osfh1 have contributed to our understanding of the genetic mechanisms underlying root growth and development [11–17]. In addition, transgenic studies have also provided evidence that several genes are involved in rice root development, such as *OsARF12*, *OsPID1*,*OsNAC6*, and *IAA3* [18–21]. These cloning of genes associated with root morphology provide a theoretical basis for root growth. However, these mutants are difficult for breeding, because most of them have obvious negative effects on grain yield or plant growth. Most agronomic traits, including those of the root, are quantitative traits. Many QTLs associated with root morphological traits have been characterized. Using different populations, more than 600 QTLs have been mapped. Champoux et al. firstly reported QTLs associated with five root parameters, including maximum root length, root dry weight per tiller, root/shoot ratio, deep root dry weight per tiller and root thickness, using the 203 recombinant inbred lines (RILs) derived from *indica* cultivar Co39 and *japonica* cultivar Moroberekan [22]. Subsequently, Price and Tomos mapped QTLs for eight root growth characteristics using an F_2_ population derived from two drought-resistant rice varieties, Bala and Azucena [23]. Yadav et al. identified QTLs related to root traits using a doubled haploid (DH) population [24]. Price et al. identified 24 regions containing QTLs for different root traits in 140 RILs derived from Bala and Azucena [25]. Venuprasad et al. tagged several QTL associated with root morphological traits from the doubled haploid population of IR64 and Azucena [26]. Courtois et al. located QTLs related to several constitutive root traits, including maximum root length, root thickness and root dry weight in various layers in 125 RILs of IAC165 and Co39 [27]. Zheng et al. mapped QTLs related to root traits and screened two candidate genes from expressed sequence tags (ESTs) and cDNA-amplification length polymorphisms (AFLP) clones [28]. Yue et al. employed 180 RILs developed from Zhenshan 97 and IRAT109, and investigated 36 QTLs for five root traits under control, and 38 for seven root traits under drought stress conditions [29]. Courtois et al. detected 51 unique loci associated with root traits adopting genome-wide association mapping using 167 *japonica* accessions [30].

So far, only four QTLs for rice root morphology have been fine mapped. *Sta1*, a QTL determining stele transversal area, was delimited in a 359-Kb interval on rice chromosome 9 using BC_2_F_3_ and BC_2_F_4_ populations [31]. *qRL6*.*1*, a major QTL for root elongation, was fine mapped into a 337-Kb region using a CSSL and its derived population [32]. *Dro1*, controlling rice deep root, was narrowed into a 608.4-Kb segment [33]. Recently, Uga et al. identified *qSOR1*, a major QTL involved in soil-surface rooting in paddy fields and located it to an 812-kb interval using seven BC_2_F_3_ recombinant lines[34]. More recently, *Dro1* was cloned into a 6-kb interval [35].

Advanced populations, such as chromosome segment substituted lines (CSSLs), have same genetic background with recurrent parent, except the donor segments. In order to analyze the genetic basis of rice root development and detecting favorable genes related with root traits, we identified 38 QTLs related to seven root traits using a set of resequencing-genotyped CSSLs under hydroponic conditions. These results provide a valuable contribution to the genetic analysis of root morphology.

## Materials and Methods

We state clearly that no specific permissions were required for these locations.

### Plant materials

A set of 128 CSSLs with a *japonica* cultivar, Nipponbare, as the donor and an *indica* cultivar, 9311, as the recurrent parent was generated as previously reported [36]. Each CSSL line was genotyped, and a high quality physical map of ultrahigh-density single nucleotide polymorphisms (SNPs) based on whole-genome re-sequencing data was constructed. Every CSSL had approximately 60,000 SNPs. On the basis of the physical locations and genotypes of these SNPs, each CSSL was genotyped, and a physical map of the 128 CSSLs was constructed [36]. In this study, the CSSLs population was employed for QTLs mapping of rice root traits and other related traits. However, the phenotypic data of three CSSLs were not obtained because of abnormal growth.

### Hydroponic culture

The experiment was carried out in Yangzhou University, Yangzhou, China (latitude 32°24' N, longitude 119°26' E) in summer in 2012. The CSSLs and their parents were grown for this experiment under hydroponic conditions, as previously described [37]. All rice seedlings were solution grown in eight concrete ponds with the inner dimensions of 880.0 cm long by 130.0 cm wide by 50 cm deep. Iron tubes at the bottom of the ponds connected the ponds to one another. The ponds were covered with concrete planks (135.0 cm long by 16.7 cm wide by 2.5 cm in deep), and each plank contained 14 holes (4 cm in diameter) for seedling fixation. Plump seeds were surface-sterilized in a 2.5% NaClO solution for 15 min, and then washed three times with distilled water. Seeds were germinated in an illuminated incubator at 30°C. Rice seedlings were transplanted from the soil seed bed 30 days after germination, and fixed into the holes with a sponge, one hole for one seedling. Fifty-six seedlings were prepared for each line in a randomized complete design with two replicates.

The nutrient solution was the mixture of the Epsino nutrient solution and the Arnon microelement nutrient solution. The mixed nutrient solution was filled into the ponds at transplanting and refreshed every 10 days. The pH value of nutrient solution was monitored daily and maintained between 5.5 and 6.5 by adding diluted H_2_SO_4_. A pump was used to keep the solution continuously cycling during the whole growth period of the rice materials, to maintain the uniformity of pH and nutrient concentration in solution ponds and to improve the O_2_ supply.

### Evaluation of phenotypes

Plants were sampled at the mature stage. Seven root traits, including root number (RN), total root length (TRL), root dry weight (RDW), maximum root length (MRL), root thickness (RTH), total absorption area (TAA) and root vitality (RV), were evaluated. For RN, only the first ramification was counted. For MRL, the length of the root was measured from the base of the plant to the tip of the longest root. TRL was the total length of the whole roots and calculated as MRL*RN/2. RTH was the average diameter of roots at the middle position of the root per plant. For RW, after deactivating enzymes at 105°C for 10 min, the roots were dried at 75°C to constant weight, and then weighed. For RV, we adopt the method of α-NA to measure root vitality [38]. The measure of TAA was by reference to the method of Xiao et al. [39].

We also measured several yield traits, including panicle number per plant (PN), number of spikelets per panicle (SPP), grain weight (GW), seed setting rate (SSR), biomass yield (BY) and grain yield (GY) during the mature stage. PN was the number of effective panicles with 10 or more grains. SPP was measured as the total number of spikelets of the whole plant divided by its total number of panicles. Grain weight was calculated on the basis of 100 grains and converted to 1000-grain weight. SSR was calculated as the number of filled grains per panicle divided by the total number of spikelets per panicle. For BY, after deactivating enzymes at 105°C for 10 min, the aboveground organ were dried at 75°C to constant weight, and then weighed. For GY, total grains per plant were dried naturally after harvesting and stored at room temperature for at least 1 month before weighing.

### QTLs analysis

QTLs analysis was conducted according to a previously published method [36]. The multiple linear model was used as the main effect model. The mean value of the *i*th line of population is defined as *y*_*i*_, the mean of the population is defined as the *b*_*0*_, in the whole genome, overall number of bins is defined as *m*, the main effect related to bin *k* is defined as *b*_*k*_, donor parent bin denotes *x*_*ik*_ = 1, recurrent parent bin denotes *x*_*ik*_ = -1. *e*_*i*_ denotes the residual error:

$$y_{i}\;=\;b_{0}+\sum_{k\;=\;1}^{m}b_{k}x_{ik}+e_{i}$$

## Results

### Phenotypic variation

**Fig 1:**
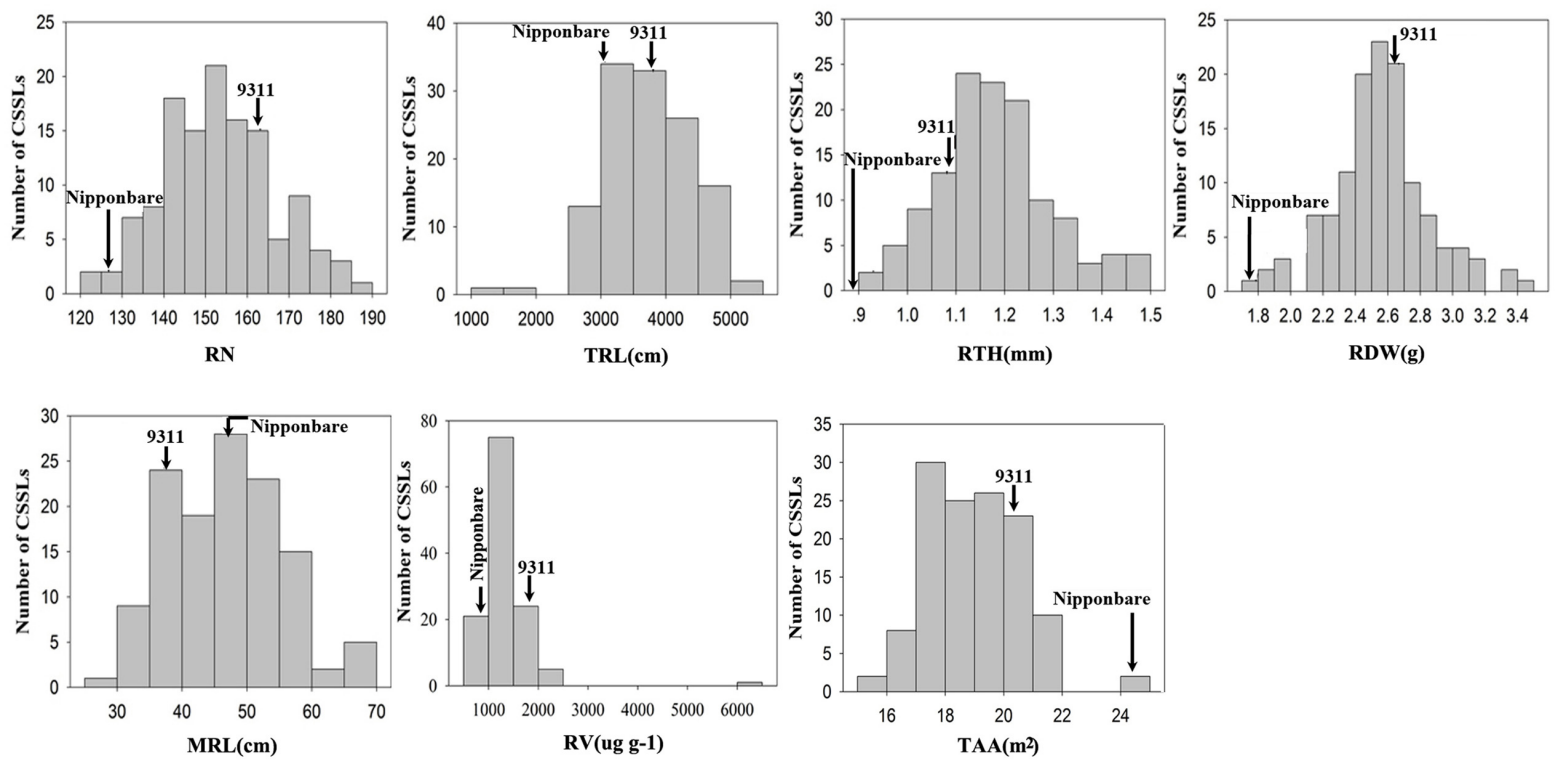
Distributions of chromosome segment substitution lines for the seven root traits.

The phenotypic value of the seven root traits of the parents and CSSL population were summarized in Table 1 and Fig 1. The two parents, 9311 and Nipponbare, showed highly significant differences in all the root traits examined. 9311 produced more, longer and heavier roots than Nipponbare (Table 1). Extensive variations and a normal distribution among CSSLs were observed for the most traits, which is in accord with the characteristics of quantitative traits (Fig 1). Except MRL, the mean phenotypic values of other six root morphology in the CSSLs population were closer to those of parent 9311 (Table 1). We can explain that the CSSLs have the 9311 genetic backgrounds.

**Table 1:** Mean values and ranges of the seven root traits in the parents and CSSLs.

Traits	CSSLs		Parents	
	Mean ± SD	Range	9311	Nipponbare
RN	153.17 ± 13.39	123.78–189.00	164.00 ± 5.69	125.67 ± 9.84
TRL (cm)	3730.69 ± 658.02	1486.67–5100.00	3800.00 ± 1652.27	3030.00 ± 984.23
RDW (g)	2.56 ± 0.30	1.79–3.43	2.65 ± 0.28	1.78 ± 0.24
RTH (mm)	1.18 ± 0.12	0.93–1.48	1.08 ± 0.08	0.93 ± 0.19
MRL (cm)	46.50 ± 8.57	29.67–69.00	37.00 ± 1.00	47.83 ± 8.80
TAA (m^2^)	19.00 ±1.62	15.50–24.73	20.90 ± 3.47	24.63 ± 5.60
RV (ug g^-1^)	1326.19 ± 554.00	532.04–6337.74	1878.91 ± 318.27	760.72 ± 110.96

#### Correlations among root morphological traits

Table 2 showed the pair-wise correlation coefficients among the seven root traits. Three among them, RN, TRL and RDW, had significant positive correlation with each other, indicating that these three traits promote each other. In addition, MRL had significant positive correlation with TRL. TRL had significant positive correlation with RDW, RTH and MRL. Positive correlation was also found between RTH and RV. Except this, a negative correlation was found between RN and MRL.

**Table 2:** Correlation coefficients among seven root traits in CSSLs.

	RN	TRL	RDW	RTH	MRL	TAA
TRL	0.240**					
RDW	0.390**	0.439**				
RTH	- 0.006	0.294**	0.213*			
MRL	- 0.249**	0.528**	0.152	- 0.060		
TAA	- 0.153	0.046	- 0.082	0.168	- 0.078	
RV	0.101	- 0.055	0.135	0.178*	- 0.062	- 0.169

* Correlation is significant at the 0.05 level (two-tailed).

** Correlation is significant at the 0.01 level (two-tailed).

We also carried out the correlation analysis between the seven root traits and yield traits (Table 3). BY had significant positive correlation with TRL, RDW and MRL, respectively. Positive correlations were also found between GY and TRL, RTH and MRL. These data suggested that these three root traits are closely related to grain and biomass yield. Positive correlations were also found between TRL and PN, RTH and SSR, GW and MRL, SSR and TAA, respectively.

**Table 3:** Correlation analysis among seven root traits and six yield-related traits in the CSSL population.

	RN	RDW	RTH	MRL	TAA	RV
PN	- 0.132	- 0.043	0.116	0.167	0.001	0.057
SPP	- 0.059	0.119	0.094	0.072	- 0.099	- 0.132
GW	0.042	0.085	0.015	0.224*	0.171	0.104
SSR	0.162	- 0.004	0.195*	0.024	0.193*	- 0.046
BY	0.017	0.220*	0.023	0.386**	- 0.090	- 0.071
GY	0.001	0.056	0.194*	0.201*	0.103	- 0.056

* Correlation is significant at the 0.05 level (two-tailed).

** Correlation is significant at the 0.01 level (two-tailed).

### QTLs identification for root morphological traits

Thirty-eight QTLs were initially detected for the seven root traits on all chromosomes, except chromosome 12.

#### TRL

Six QTLs controlling TRL were finally detected. These QTLs were *qTRL2*.*1*, *qTRL5*.*1*, *qTRL6*.*1*, *qTRL8*.*1*, *qTRL10*.*1* and *qTRL11*.*1*, which were located in X_73_ on chromosome 2, X_205_ on chromosome 5, X_242_ on chromosome 6, X_292_ on chromosome 8 X_347_ on chromosome 10 and X_388_ on chromosome 11, respectively. The QTL with the largest effect was mapped to X_388_ and occupied to the physical position of 26,318,602 bp to 30,828,668 bp (Table 4).

**Table 4:** QTLs mapping of TRL.

Bins	QTLs	Interval / bp	Size of the interval / bp	Chr.	Partial R-Square	F value
X_73_	*qTRL2*.*1*	19526907–19710693	183786	2	5.88%	9.79
X_205_	*qTRL5*.*1*	19013592–19052307	38715	5	6.86%	10.67
X_242_	*qTRL6*.*1*	21140686–22187608	1046922	6	6.24%	11.27
X_292_	*qTRL8*.*1*	11500666–14235575	2734909	8	4.18%	8.66
X_347_	*qTRL10*.*1*	17928505–18055332	126827	10	5.37%	10.45
X_388_	*qTRL11*.*1*	26318602–30828668	4510066	11	14.01%	20.21

#### RDW

Six QTLs associated with RDW were located on chromosomes 3, 6, 8 and 11, respectively. These QTLs were *qRDW3*.*1*, *qRDW6*.*1*, *qRDW8*.*1*, *qRDW8*.*2*, *qRDW8*.*3*, *qRDW11*.*1*, which were located in X_137_ on chromosome 3, X_233_ on chromosome 6, X_278_ on chromosome 8, X_284_ on chromosome 8, X_286_ on chromosome 8 and X_377_ on chromosome 11, respectively. The QTL with the largest effect was mapped to X_233_ and occupied to the physical position of 7,814,673 bp to 9,668,398 bp (Table 5).

**Table 5:** QTLs mapping of RDW.

Bins	QTLs	Interval / bp	Size of the interval / bp	Chr.	Partial R-Square	F value
X_137_	*qRDW3*.*1*	22449287–22467112	17825	3	6.15%	10.78
X_233_	*qRDW6*.*1*	7814673–9668398	1853725	6	9.15%	12.49
X_278_	*qRDW8*.*1*	2797908–3336084	538176	8	7.37%	11.96
X_284_	*qRDW8*.*2*	6251746–7203791	952045	8	8.27%	12.31
X_286_	*qRDW8*.*3*	7817482–8945724	1128242	8	4.22%	8.39
X_377_	*qRDW11*.*1*	7416940–10601700	3184760	11	5.01%	9.39

#### MRL

Similarly, we detected eight QTLs controlling MRL under hydroponic conditions. These QTLs were *qMRL5*.*1*, *qMRL6*.*1*, *qMRL7*.*1*, *qMRL8*.*1*, *qMRL9*.*1*, *qMRL9*.*2*, *qMRL10*.*1* and *qMRL11*.*1* which were located in X_207_ on chromosome 5, X_233_ on chromosome 6, X_262_ on chromosome 7, X_291_ on chromosome 8, X_316_ on chromosome 9, X_318_ on chromosome 9 X_352_ on chromosome 10 and X_388_ on chromosome 11, respectively. The QTL with the largest effect was mapped to X_207_ and occupied to the physical position of 19,287,670 bp to 19,403,538 bp (Table 6).

**Table 6:** QTLs mapping of MRL.

Bins	QTLs	Interval / bp	Size of the interval / bp	Chr.	Partial R-Square	F value
X_207_	*qMRL5*.*1*	19287670–19403538	115868	5	9.22%	12.59
X_233_	*qMRL6*.*1*	7814673–9668398	1853725	6	3.42%	6.98
X_262_	*qMRL7*.*1*	8527898–8572465	44567	7	4.68%	8.61
X_291_	*qMRL8*.*1*	90684529–11500666	1816137	8	6.67%	9.76
X_316_	*qMRL9*.*1*	6840683–7638718	798035	9	7.06%	12.21
X_318_	*qMRL9*.*2*	9688069–11709751	2021682	9	3.82%	8.26
X_352_	*qMRL10*.*1*	18855550–20084853	1229303	10	7.13%	11.29
X_388_	*qMRL11*.*1*	26318602–30828668	4510066	11	3.95%	7.66

#### RN

Seven QTLs for RN were detected on chromosomes 1, 3, 5, 6, 9 and 11, respectively. These QTLs were *qRN1*.*1*, *qRN3*.*1*, *qRN3*.*2*, *qRN5*.*1*, *qRN6*.*1*, *qRN9*.*1* and *qRN11*.*1*, which were located in X_8_ on chromosome 1, X_131_ on chromosome 3, X_139_ on chromosome 3, X_221_ on chromosome 5, X_241_ on chromosome 6, X_321_ on chromosome 9 and X_380_ on chromosome 11, respectively. The QTL with the largest effect was mapped to X_207_ and occupied to the physical position of 19,120,157 bp to 19,494,142 bp (Table 7).

**Table 7:** QTLs mapping of RN.

Bins	QTLs	Interval / bp	Size of the interval / bp	Chr.	Partial R-Square	F value
X_8_	*qRN1*.*1*	12001587–12038149	36562	1	6.31%	8.96
X_131_	*qRN3*.*1*	15131566–15146848	15282	3	3.62%	6.95
X_139_	*qRN3*.*2*	23156128–24417950	1261822	3	6.36%	9.68
X_221_	*qRN5*.*1*	25834805–26310200	475395	5	5.40%	9.34
X_241_	*qRN6*.*1*	20704751–21140686	435935	6	5.39%	8.71
X_321_	*qRN9*.*1*	14738433–15449101	710668	9	4.29%	7.85
X_380_	*qRN11*.*1*	19120157–19494142	373985	11	7.14%	9.53

#### RTH

Four putative QTLs associated with RTH were detected on chromosomes 1, 2, 5 and 10, respectively. These QTLs were *qRTH1*.*1*, *qRTH2*.*1*, *qRTH5*.*1*, and *qRTH10*.*1*, which were located in X_24_ on chromosome 1, X_64_ on chromosome 2, X_193_ on chromosome 5 and X_347_ on chromosome 10, respectively. The QTL with the largest effect was mapped to X_347_ and occupied to the physical position of 17,928,505 bp to 18,055,332 bp (Table 8).

**Table 8:** QTLs mapping of RTH.

Bins	QTLs	Interval / bp	Size of the interval / bp	Chr.	Partial R-Square	F value
X_24_	*qRTH1*.*1*	34259334–35734160	1474826	1	5.12%	7.71
X_64_	*qRTH2*.*1*	11015128–11144160	129032	2	6.75%	9.64
X_193_	*qRTH5*.*1*	2208395–3519762	1311367	5	4.47%	7.07
X_347_	*qRTH10*.*1*	17928505–18055332	126827	10	7.14%	9.53

#### TAA

Two QTLs for TAA were detected on chromosomes 2 and 11, respectively. These QTLs were *qTAA2*.*1* and *qTAA11*.*1*, which were located in X_66_ on chromosome 2 and X_387_ on chromosome 11, respectively. The QTL with the largest effect was mapped to X_387_ and occupied to the physical position of 25,559,185 bp to 26,317,711 bp (Table 9).

**Table 9:** QTLs mapping of TAA.

Bins	QTLs	Interval / bp	Size of the interval / bp	Chr.	Partial R-Square	F value
X_66_	*qTAA2*.*1*	16341872–17154017	812145	2	6.74%	9.96
X_387_	*qTAA11*.*1*	25559185–26317711	758526	11	10.06%	13.86

#### RV

Five putative QTLs associated with RV were detected on chromosomes 3, 4 and 5 respectively. These QTLs were *qRV3*.*1*, *qRV4*.*1*, *qRV5*.*1*, *qRV5*.*2* and *qRV5*.*3*, which were located in X_134_ on chromosome 3, X_160_ on chromosome 4 and X_218_, X_219_, X_225_ on chromosome 5, respectively. The QTL with the largest effect was mapped to X_160_ and occupied to the physical position of 16,578,162 bp to 26,317,711 bp (Table 10).

**Table 10:** QTLs mapping of RV.

Bins	QTLs	Interval / bp	Size of the interval / bp	Chr.	Partial R-Square	F value
X_134_	*qRV3*.*1*	16578162–16634855	56693	3	2.23%	8.58
X_160_	*qRV4*.*1*	4886858–15540893	10654035	4	37.08%	73.06
X_218_	*qRV5*.*1*	23976183–24154129	177946	5	16.66%	60.49
X_219_	*qRV5*.*2*	24154129–25095306	941177	5	5.80%	14.15
X_225_	*qRV5*.*3*	28689993–29833991	1143998	5	7.12%	15.69

Among these QTLs, we found that some bins controlling one trait was simultaneously detected for other traits. *qTRL10*.*1* and *qRTH10*.*1* were simultaneously located in bin X_347_. *qTRL11*.*1* and *qMRL11*.*1* were detected in X_388_. *qRDW6*.*1* and *qMRL6*.*1* were detected in X_233_.

## Discussion

Rice has the greatest water requirement, compared with other cereals [40].Rice production also needs a supply of exogenous nutrients, especially nitrogen fertilizer [41]. Rice roots play an important role in water and nutrient uptake [42]. More water and nutrient uptake of rice depend on faster and more extensive root growth. [43]. Additionally, some studies found that rice root traits have positive relationship with grain yield. [44]. Among different rice varieties, there are large variation associated with root traits.[45]. Generally speaking, *indica* rice varieties have fewer roots, smaller root absorbing areas, lower root absorbing area density, lower root/shoot ratio, larger root volume and root length, and show an earlier decline in root parameters than *japonica* rice variety. The upland rice variety has a greater rooting capacity, root length, root absorbing area density and root/shoot ratio [46].

The development of crop cultivars with thicker and deeper roots is expected to increase water and nutrient availability. Therefore, improving our understanding of the genetics of rice roots could have a significant impact on yield production and water saving. Most of the root traits showed polygenic inheritance. A more thorough knowledge of genetic mechanism of root traits is very important for root breeding program, and there are few examples of the introduction in breeding programs of root traits [5,42,47]. Although hundreds of QTLs associated with rice root traits have been found by different populations, including F_2_, DHs, and mostly RILs, only four QTLs have been fine mapped.

Primary populations such as F_2_, DHs and RILs are very easy to develop; however, due to their genetic background noise, they are difficult for further study [48]. Therefore, advanced mapping populations, like CSSLs, have been developed. CSSLs have same genetic background with female, except the donor segments. Thus, CSSLs make QTLs analysis easier than before and many QTLs of important agronomic traits have been detected in this way [36,49–55]. In our previous work, we developed a set of 128 CSSLs generated using an *indica* cultivar, 9311, as the recurrent and a *japonica* cultivar, Nipponbare, as the donor. We detected accurately the lengths of the substituted segments and provided more accurate background information using the resequencing-based map [36].

**Fig 2:**
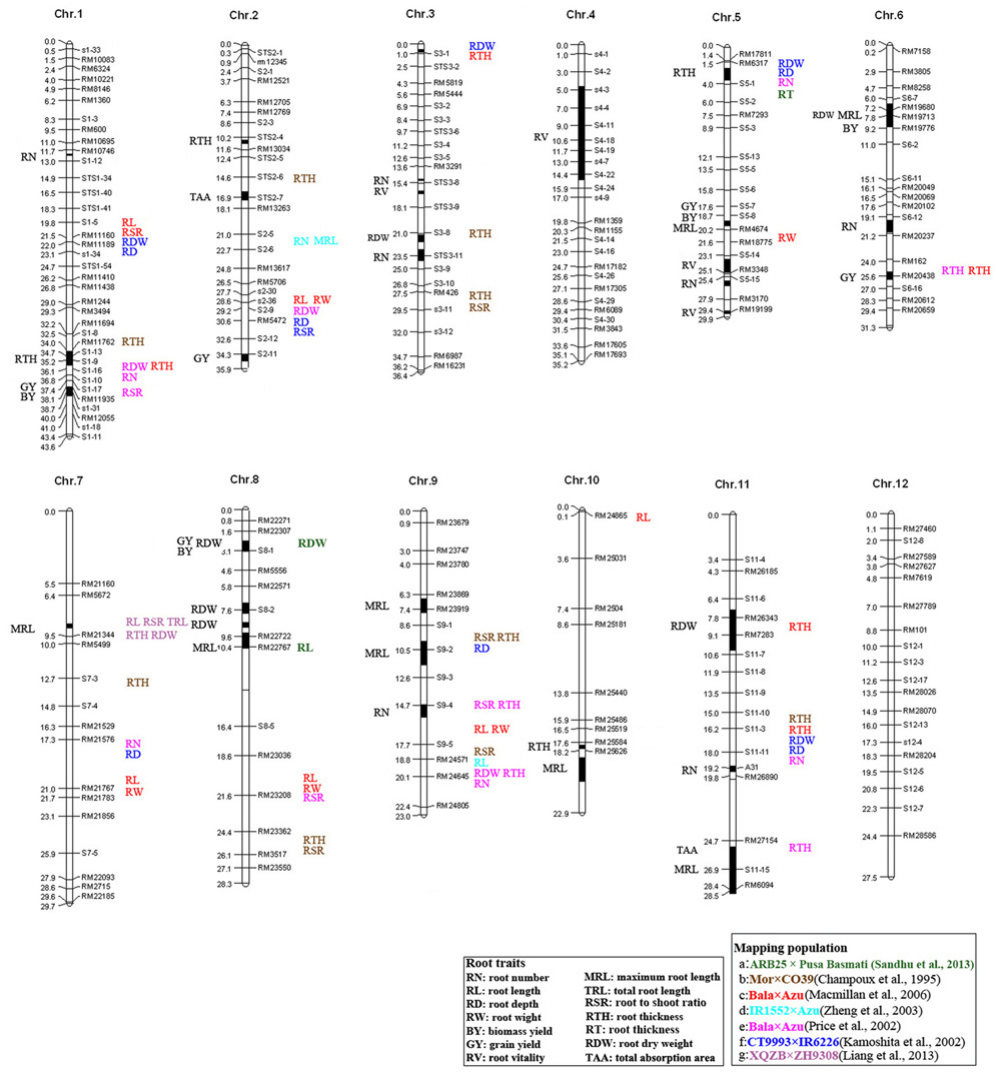
Bin allocations on the rice molecular linkage map of the QTLs identified in this study and other nine mapping populations evaluated for root traits. Acronyms on the left of the chromosomes represent QTLs identified in this study and acronyms to the right of the chromosomes represent QTLs identified in the other nine mapping populations. Vertical parentheses spanning two bins indicate QTLs that could not be assigned to a single bin. The colors of acronyms for root traits represent the nine populations in which such traits were identified.

In this study, 38 QTLs associated with root morphology were identified using the CSSLs population mentioned above. Among these QTLs, *qRV4*.*1* explained the largest variance of phenotypic effect (37.08%). *qMRL6*.*1* explained the least variance of phenotypic effect (3.42%). Four QTLs identified for all root traits was found for more than 10% phenotypic variation explained by a single QTL. Several of them were located on the same chromosome segment reported in previous studies (Fig 2). For example, *qRTH1*.*1* was on bin X_24_ close to the QTL for maximum root thickness detected in the Bala × Azu cross [56]. *qRN6*.*1* was identified on bin X_241_ close to the QTL for lateral root number detected in the IR1552 × Azu cross [28]. *qMRL7*.*1*, identified on bin X_262_, was locating on a overlapping region containing a pleiotropic QTL for seven root traits on chromosome 7 [57]. *qRN9*.*1*, locating on bin X_321_, was very close to a fine mapped QTL, *Sta1*, which determines the stele transversal area on chromosome 9 [31]. Additionally, *qMRL8*.*1* on bin X_291_, *qRTH5*.*1* on X_193_ and *qRDW8*.*1* on X_278_ co-localized on the same region with *qRL8*.*1*, *qRT5*.*1* and *qRDW8*.*1*, associated with root length, root thickness and dry root weight, respectively, using an ARB25 × Pusa Basmati 1460 F_2:3_ population [58].

A well-developed root system increases biomass and yield in different treatments and cultivars in paddy fields [6]. We are interested in whether or not the QTLs for root traits are located on the overlapping region containing QTLs regulating grain yield and biomass. Actually, several QTLs associated with grain yield and biomass yield found in other studies also close to the bins controlling root traits found in our research. For example, *qMRL11*.*1* was located on the chromosome segment containing a QTL for shoot biomass detected in a DH population from the cross of CT9993 × IR62266 [9]. *qRTH1*.*1* on bin X_24_ and *qRDW3*.*1* on X_137_ had the similar positions with two QTLs, *yd1b* and *yd3*, respectively, which were involved in grain yield found in RILs from the cross of Zhenshan97 and Minghui63 [59]. Both of *qRV4*.*1* on bin X_160_ and *qYLD4*-1, a QTL for grain yield per plant identified in the DH population from IR64 and Azucena [60], was located in a overlapping region. In addition, *qRTH2*.*1* on bin X_64_ shared same region with two QTLs, *DTY2*.*1* [61] and *qDTY2*.*1* [62], regulating grain yield under drought conditions. This result implied that *qRTH2*.*1* maybe a valuable loci to improve the ability to avoid drought stress and beneficial for stable rice production.

In this study, a total of 38 QTLs associated with root morphology were identified using a resequencing-genotyped CSSLs population under hydroponic culture conditions. Six of the detected QTLs were narrowed in very small regions. For instance, *qTRL5*.*1* on bin X_205_, *qRDW3*.*1* on bin X_137_, *qMRL7*.*1* on bin X_262_, *qRN1*.*1* on bin X_8_, *qRN3*.*1* on bin X_131_, and *qRV3*.*1* on bin X_134_ were delimited into chromosome segments less than 100-Kb in physical distance. These results would be helpful for cloning of these QTLs and improving rice water and fertilizer use efficiency by molecular breeding of root system architectures.

## Supporting Information

S1 File Original data of root traits. (XLS)

## Abbreviations

BY: biomass yield
CSSLs: chromosome segment substitution lines
DHs: doubled haploid lines
EST: expressed sequence tag
GW: grain weight
GY: grain yield
NILs: near isogenic lines
MAS: Marker-assisted selection
MRL: maximum root length
PN: panicle number per plant
QTLs: quantitative trait loci
RDW: root dry weight
RILs: recombinant inbred lines
RN: root number
RSR: root to shoot ratio
RTH: root thickness
RV: root vitality
SNPs: single nucleotide polymorphisms
SPP: number of spikelets per panicle
SRR: shoot to root ratio
SSR: seed setting rate
STS: sequence tagged site
TRL: total root length
TAA: total absorption area
