# Correction: Mapping Quantitative Trait Loci Associated with Toot Traits Using Sequencing-Based Genotyping Chromosome Segment Substitution Lines Derived from 9311 and Nipponbare in Rice (*Oryza sativa* L.)

**DOI:** 10.1371/journal.pone.0155280

**Published:** 2016-05-04

**Authors:** Yong Zhou, Guichun Dong, Yajun Tao, Chen Chen, Bin Yang, Yue Wu, Zefeng Yang, Guohua Liang, Baohe Wang, Yulong Wang

There are errors in the title. The correct title is: Mapping Quantitative Trait Loci Associated with Root Traits Using High Throughput Genotyped Chromosome Segment Substitution Lines Derived from 9311 and Nipponbare in Rice (*Oryza sativa* L.). The correct citation is: Zhou Y, Dong G, Tao Y, Chen C, Yang B, Wu Y, et al. (2016) Mapping Quantitative Trait Loci Associated with Root Traits Using High Throughput Genotyped Chromosome Segment Substitution Lines Derived from 9311 and Nipponbare in Rice (*Oryza sativa* L.). PLoS ONE 11(3): e0151796. doi:10.1371/journal.pone.0151796.

In Table 3, the column “TRL” is missing. Please see the corrected Table 3 here.

**Table 3:** Correlation analysis among seven root traits and six yield-related traits in the CSSL population.

	RN	TRL	RDW	RTH	MRL	TAA	RV
PN	- 0.132	0.194*	- 0.043	0.116	0.167	0.001	0.057
SPP	- 0.059	0.003	0.119	0.094	0.072	- 0.099	- 0.132
GW	0.042	0.137	0.085	0.015	0.224*	0.171	0.104
SSR	0.162	0.138	- 0.004	0.195*	0.024	0.193*	- 0.046
BY	0.017	0.194*	0.220*	0.023	0.386**	- 0.090	- 0.071
GY	0.001	0.209*	0.056	0.194*	0.201*	0.103	- 0.056

* Correlation is significant at the 0.05 level (two-tailed).

** Correlation is significant at the 0.01 level (two-tailed).
